# Supplementary material for: Temperature-resilient anapole modes associated with TE polarization in semiconductor nanowires
Source: Sci Rep. 2022 Dec 9;12:21345. doi: 10.1038/s41598-022-25289-w (PMC9734189; doi:10.1038/s41598-022-25289-w)
Supplement: Supplementary file 1 — Supplementary Information. [file 41598_2022_25289_MOESM1_ESM.pdf]

Supporting Information:

Temperature-resilient anapole modes associated with TE polarization in semiconductor nanowires

Vaibhav Thakore[1,*], Tapio Ala-Nissila[2,3,4,*], and Mikko Karttunen[5,6,7,*]

[1]Department of Applied Mathematics, Western University, 1151 Richmond Street, London, Ontario N6A 5B7, Canada
[2]QTF Center of Excellence, Department of Applied Physics, Aalto University School of Science, FIN-00076, Aalto, Espoo, Finland
[3]Department of Physics, Brown University, Providence, Rhode Island 02912-1843, USA
[4]Interdisciplinary Centre for Mathematical Modelling, Department of Mathematical Sciences, Loughborough University, Loughborough LE11 3TU, UK
[5]Department of Physics and Astronomy, Western University, 1151 Richmond Street, London, Ontario N6A 3K7, Canada
[6]The Centre of Advanced Materials and Biomaterials Research, Western University, 1151 Richmond Street, London, Ontario, N6A 5B7, Canada
[7]Department of Chemistry, Western University, 1151 Richmond Street, London, Ontario, N6A 5B7, Canada
[*]vthakore@knights.ucf.edu, tapio.ala-nissila@aalto.fi and mkarttu@uwo.ca

The Supporting Information includes: four figures S1-S4 that show the scattering and absorption efficiencies for Au, Si and GaAs as a function of the nanowire radius r and wavelength λ of the incident EM radiation at temperatures $T = 200$, 470, and $650\,\mathrm{K}$ for the TM and TE polarizations; and, six tables S1-S6 that summarize the spectral positions of the resonances, the contributing orders to the scattering and absorption resonances and the values of the scattering and absorption efficiencies for both perpendicular and parallel polarizations of the incident radiation at temperatures $T = 200$ and $650\,\mathrm{K}$.

Note: In Tables S1-S6, under the column "Order of resonance n", the entry "joint" refers to a resonance that has contributions from multiple orders (≤ 2) and "other" refers to a resonance that includes contributions from resonance order/s greater than 2.

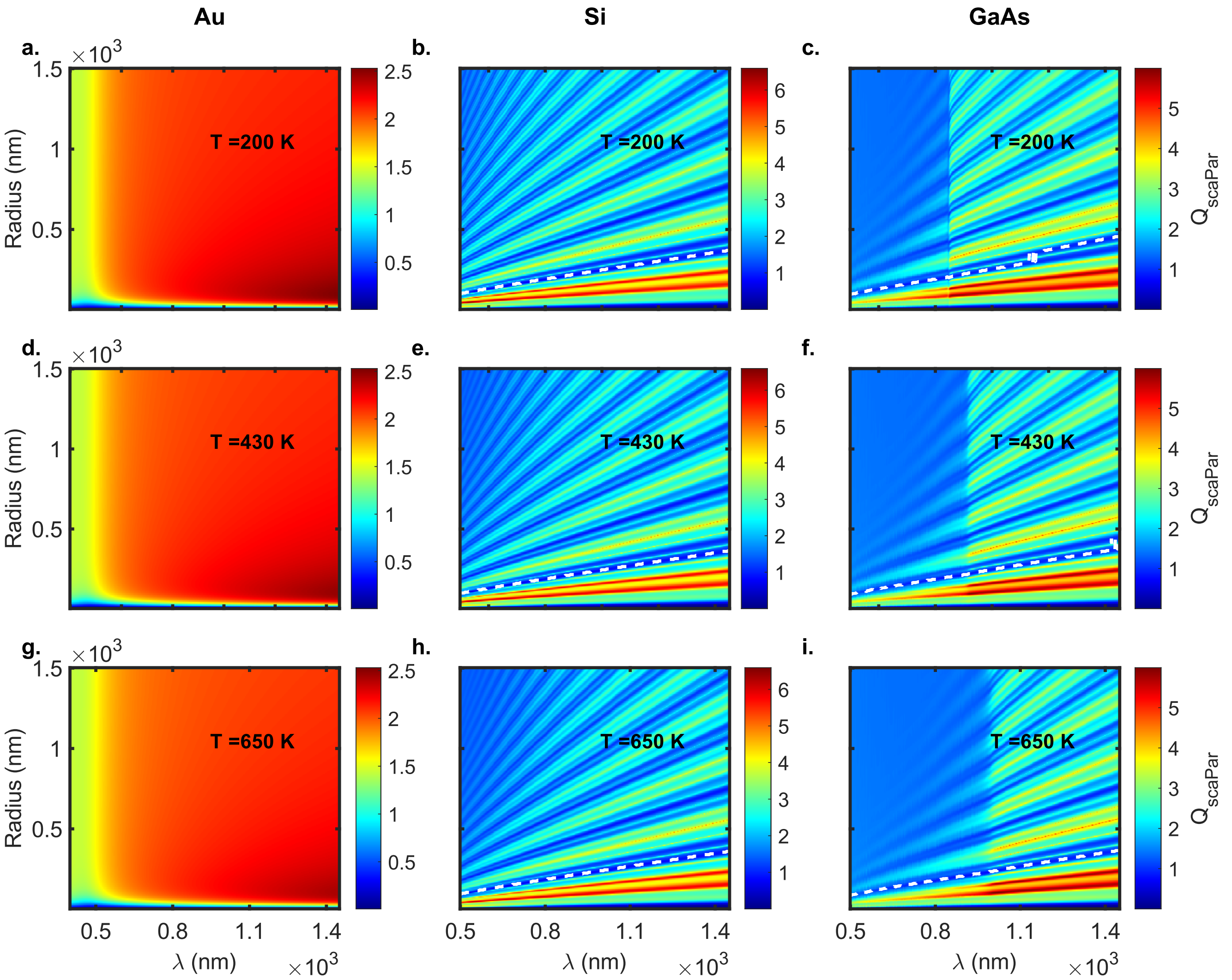

Figure S1: Mie scattering efficiency, Q_{scaPar}, for the TM ($E_{\|}$) polarization of the incident radiation as a function of the wavelength, λ, of the incident radiation and the nanowire radii r at three different temperatures: $T = 200$ (a-c), 430 (d-f) and 650 K (g-i) for Au (DL model), Si and GaAs nanowires, respectively. (See Figure 1 also.) The white dashed lines in sub-plots for Si (b, e, h) and GaAs (c, f, i) nanowires show the trajectory of the minima in Q_{scaPar} as a function of the nanowire radii ($r > 50\,\mathrm{nm}$) and wavelength λ of the incident radiation.

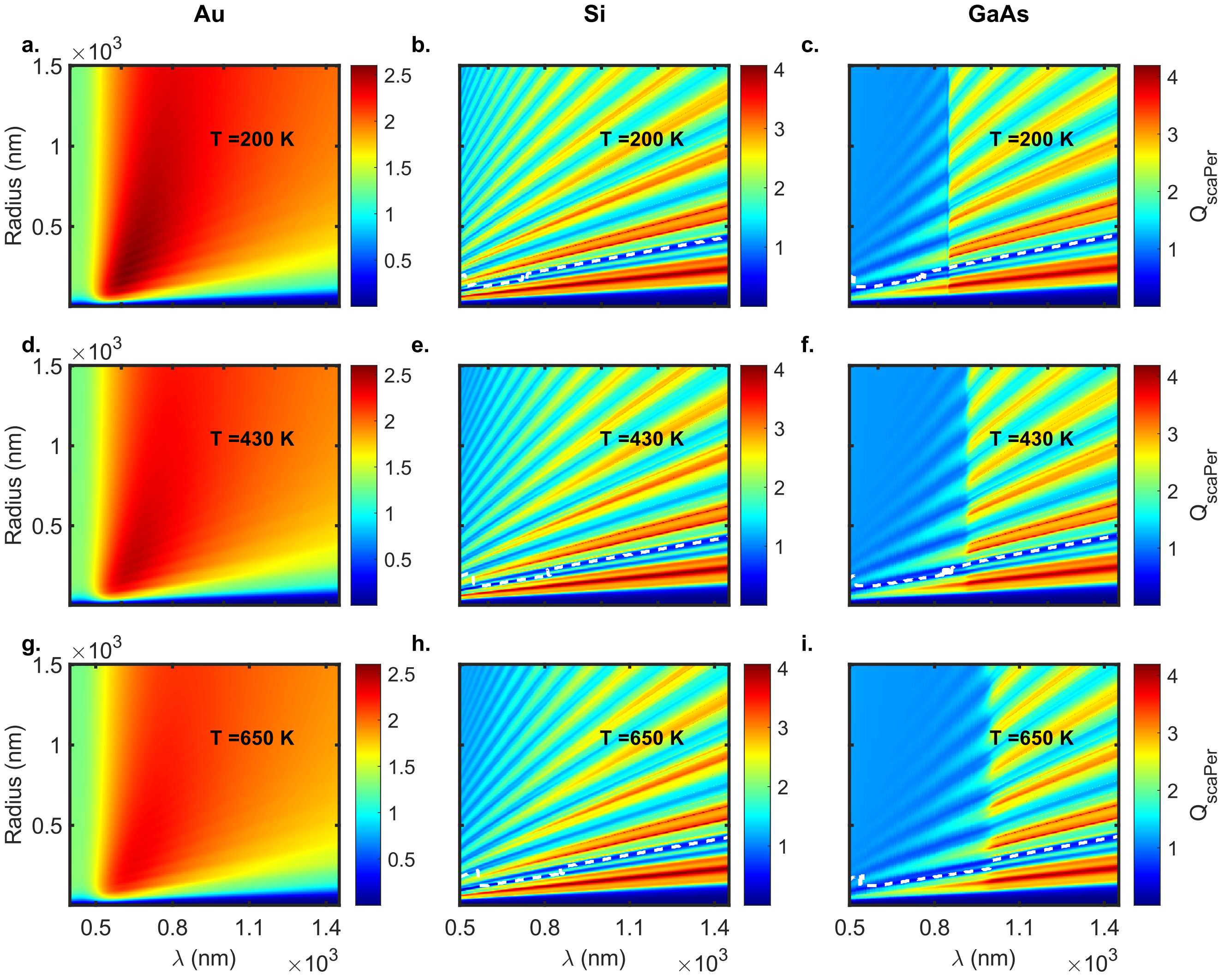

Figure S2: Mie scattering efficiency, Q_{scaPer}, for the TE ($E_{\perp}$) polarization of the incident radiation as a function of the wavelength, λ, of the incident radiation and the nanowire radii r at three different temperatures: $T = 200$ (a-c), 430 (d-f) and 650 K (g-i) for Au (DL model), Si and GaAs nanowires, respectively. (See Figure 2 also.) The white dashed lines in sub-plots for Si (b, e, h) and GaAs (c, f, i) nanowires show the trajectory of the minima in Q_{scaPer} as a function of the nanowire radii ($r > 125\,\mathrm{nm}$) and wavelength λ of the incident radiation.

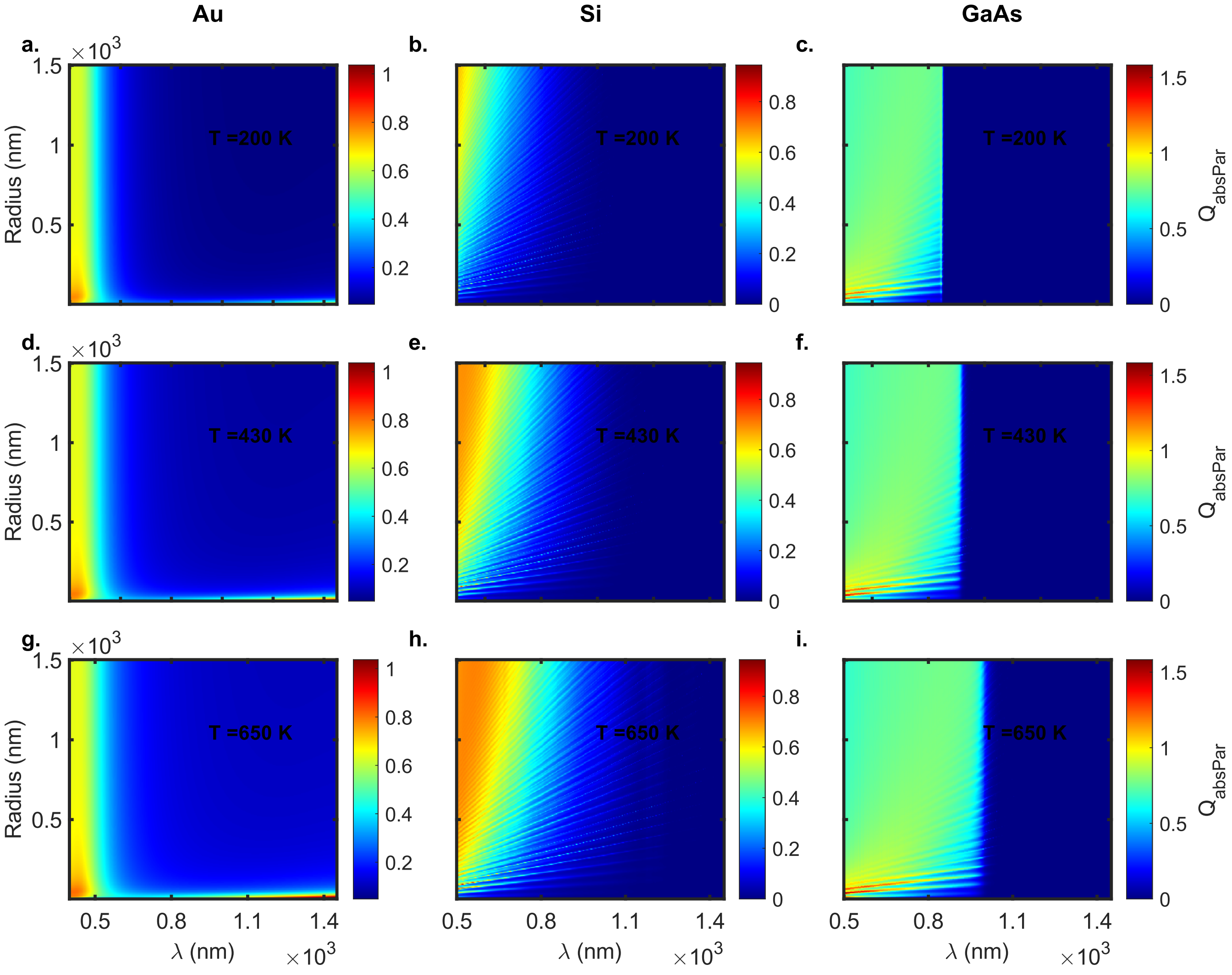

Figure S3: Mie absorption efficiency, Q_{absPar}, for the TM ($E_{\|}$) polarization of the incident radiation as a function of the wavelength, λ, of the incident radiation and the nanowire radii r at three different temperatures $T = 200$ (a-c), 430 (d-f) and 650 K (g-i) for Au (DL model), Si and GaAs nanowires, respectively. (See Figure 3 also.)

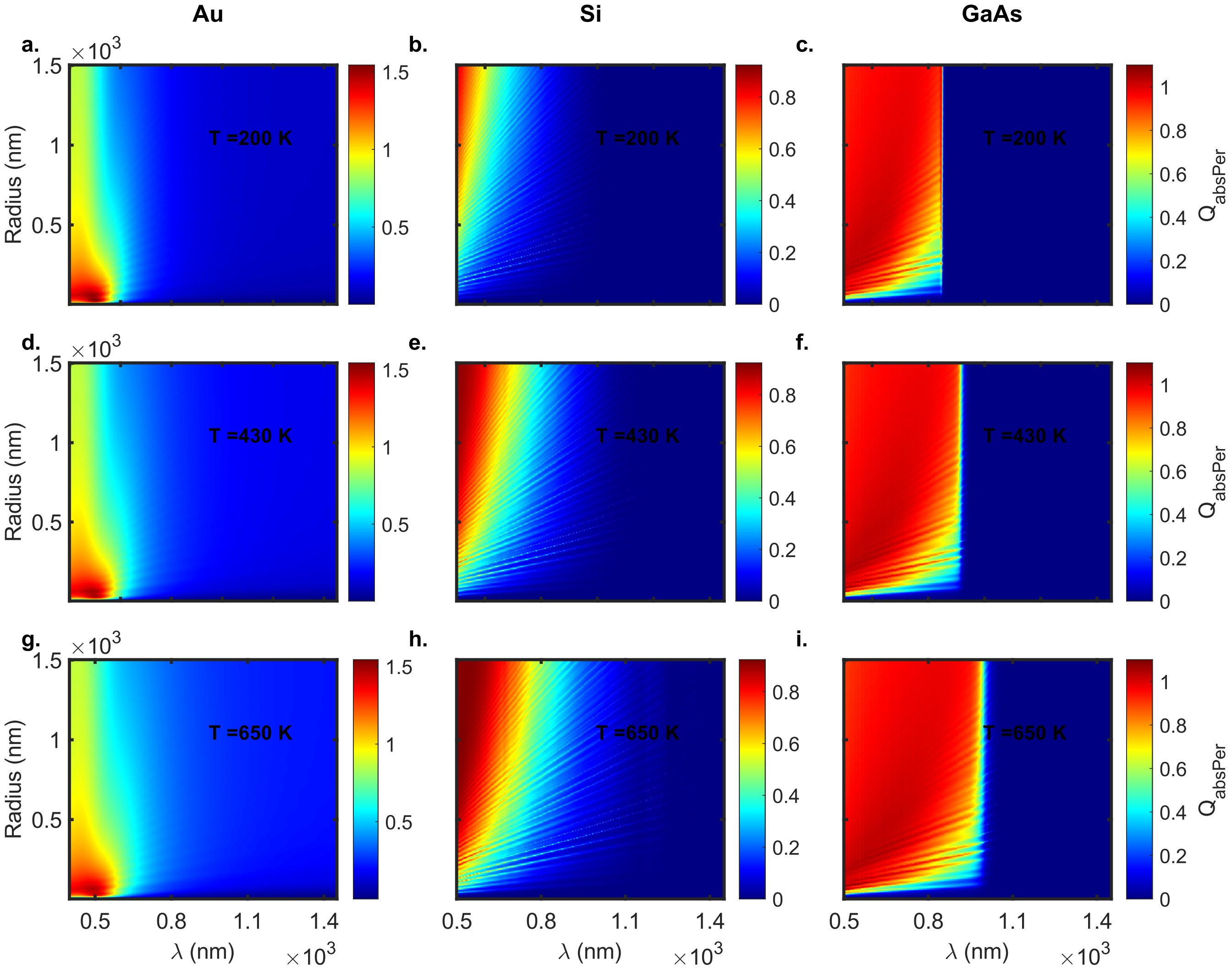

Figure S4: Mie absorption efficiency, Q_{absPer}, for the TE ($E_{\perp}$) polarization of the incident radiation as a function of the wavelength, λ, of the incident radiation and the nanowire radii r at three different temperatures $T = 200$ (a-c), 430 (d-f) and 650 K (g-i) for Au (DL model), Si and GaAs nanowires, respectively. (See Figure 4 also.)

Table S1a: Au nanowires - Scattering resonances for parallel polarization

Nanowire radius r (nm)	Order of resonance n	Temperature, $T = 200$ K			Temperature, $T = 650$ K		
		Resonance wavelength $\lambda_{\text{res}}^{\text{sca}}$ (nm)	Scattering efficiency Q_{scaPar}	Absorption efficiency Q_{absPar}	Resonance wavelength $\lambda_{\text{res}}^{\text{sca}}$ (nm)	Scattering efficiency Q_{scaPar}	Absorption efficiency Q_{absPar}
20	zeroth	1450	1.068	0.5095	1450	0.980	0.8923
80	zeroth	1450	2.511	0.1287	1450	2.397	0.2606
140	zeroth	1450	2.506	0.0906	1450	2.412	0.1878
	first	626	1.971	0.1739	633	1.893	0.2586
	second	400	1.385	0.7051	400	1.383	0.7201
200	zeroth	1450	2.450	0.0765	1450	2.368	0.1599
	first	878	2.223	0.07625	888	2.149	0.1542
	second	557	1.840	0.2736	560	1.764	0.3572

Table S1b: Au nanowires - Scattering resonances for perpendicular polarization

Nanowire radius r (nm)	Order of resonance n	Temperature, $T = 200$ K			Temperature, $T = 650$ K		
		Resonance wavelength $\lambda_{\text{res}}^{\text{sca}}$ (nm)	Scattering efficiency Q_{scaPer}	Absorption efficiency Q_{absPer}	Resonance wavelength $\lambda_{\text{res}}^{\text{sca}}$ (nm)	Scattering efficiency Q_{scaPer}	Absorption efficiency Q_{absPer}
20	Joint	528	0.412	1.162	532	0.320	1.074
80	Joint	566	2.284	0.9775	576	1.968	1.049
140	Joint	593	2.550	0.7265	605	2.235	0.8490
200	Joint	613	2.596	0.5942	625	2.293	0.7365

Table S2a: Si nanowires - Scattering resonances for parallel polarization

Nanowire radius r (nm)	Order of resonance n	Temperature, $T = 200$ K			Temperature, $T = 650$ K		
		Resonance wavelength $\lambda_{\text{res}}^{\text{sca}}$ (nm)	Scattering efficiency Q_{scaPar}	Absorption efficiency Q_{absPar}	Resonance wavelength $\lambda_{\text{res}}^{\text{sca}}$ (nm)	Scattering efficiency Q_{scaPar}	Absorption efficiency Q_{absPar}
20	zeroth	500	4.142	0.0944	500	4.294	0.2499
80	zeroth	1346	3.057	2.132e-14	1399	3.176	4.731e-5
	first	769	5.979	0.03319	791	6.008	0.10765
	second	556	5.426	0.2707	572	4.630	0.6479
140	first	1272	5.660	5.189e-11	1304	5.808	7.225e-4
	second	876	5.781	0.0212	898	5.646	0.1073
200	first	922	3.717	0.01901	944	3.483	0.1233
	second	1216	5.768	1.192e-8	1242	5.797	4.285e-3

Table S2b: Si nanowires - Scattering resonances for perpendicular polarization

Nanowire radius r (nm)	Order of resonance n	Temperature, $T = 200$ K			Temperature, $T = 650$ K		
		Resonance wavelength $\lambda_{\text{res}}^{\text{sca}}$ (nm)	Scattering efficiency Q_{scaPer}	Absorption efficiency Q_{absPer}	Resonance wavelength $\lambda_{\text{res}}^{\text{sca}}$ (nm)	Scattering efficiency Q_{scaPer}	Absorption efficiency Q_{absPer}
20	zeroth	500	0.0873	5.583e-3	500	0.0925	1.604e-2
80	zeroth	750	2.949	1.923e-2	773	2.915	6.0115e-2
	first	568	3.898	0.1069	590	3.585	0.2586
140	zeroth	1220	2.892	2.540e-9	1258	2.923	4.750e-4
	first	890	4.007	8.887e-3	916	3.950	4.558e-2
	second	714	3.002	4.802e-2	734	2.808	0.1481
200	first	1232	4.008	2.053e-9	1262	4.017	6.592e-4
	second	980	3.148	2.155e-3	1003	3.065	3.015e-2
	other	786	1.968	5.172e-2	804	1.862	0.1766

Table S3a: GaAs nanowires - Scattering resonances for parallel polarization

Nanowire radius r (nm)	Order of resonance n	Temperature, $T = 200$ K			Temperature, $T = 650$ K		
		Resonance wavelength λ_{res}^{sca} (nm)	Scattering efficiency Q_{scaPar}	Absorption efficiency Q_{absPar}	Resonance wavelength λ_{res}^{sca} (nm)	Scattering efficiency Q_{scaPar}	Absorption efficiency Q_{absPar}
20	zeroth	500	3.31	0.7956	500	3.275	0.9693
80	zeroth	1288	2.915	1e-16	1349	3.114	1.062e-6
	first	774	5.051	0.5092	824	4.757	0.7561
140	first	1234	5.480	8.882e-16	1292	5.746	6.497e-6
	second	872	5.814	2.594e-5	926	4.264	0.7465
200	second	1188	5.720	3.553e-15	1236	5.793	2.552e-5
	other	914	3.772	4.25e-9	978	2.362	0.4935

Table S3b: GaAs nanowires **-** Scattering resonances for perpendicular polarization

Nanowire radius r (nm)	Order of resonance n	Temperature, $T = 200$ K			Temperature, $T = 650$ K		
		Resonance wavelength λ_{res}^{sca} (nm)	Scattering efficiency Q_{scaPer}	Absorption efficiency Q_{absPer}	Resonance wavelength λ_{res}^{sca} (nm)	Scattering efficiency Q_{scaPer}	Absorption efficiency Q_{absPer}
20	zeroth	500	0.0925	6.537e-2	500	0.1022	9.950e-2
80	zeroth	746	2.487	0.2910	774	2.288	0.4144
	first	612	2.53	0.5976	654	2.343	0.5891
140	first	1172	2.854	1.332e-15	1244	2.913	6.186e-6
	second	884	4.020	2.139e-7	988	3.477	0.1227
200	first	1199	3.998	1.332e-15	1254	4.014	1.002e-5
	second	970	3.181	5.38e-11	1018	3.053	2.095e-2

Table S4a: Au nanowires - Absorption resonances for parallel polarization

Nanowire radius r (nm)	Order of resonance n	Temperature, $T = 200$ K			Temperature, $T = 650$ K		
		Resonance wavelength λ_{res}^{abs} (nm)	Absorption efficiency Q_{absPar}	Scattering efficiency Q_{scaPar}	Resonance wavelength λ_{res}^{abs} (nm)	Absorption efficiency Q_{absPar}	Scattering efficiency Q_{scaPar}
20	zeroth	410	0.7514	0.3463	414	0.7918	0.3644
	zeroth	1450	0.5095	1.068	1450	0.8923	0.9803
80	zeroth	421	0.7511	1.207	426	0.7753	1.208
140	zeroth	425	0.7105	1.370	429	0.7292	1.366
200	zeroth	426	0.6882	1.417	430	0.7045	1.410

Table S4b: Au nanowires - Absorption resonances for perpendicular polarization

Nanowire radius r (nm)	Order of resonance n	Temperature, $T = 200$ K			Temperature, $T = 650$ K		
		Resonance wavelength λ_{res}^{abs} (nm)	Absorption efficiency Q_{absPer}	Scattering efficiency Q_{scaPer}	Resonance wavelength λ_{res}^{abs} (nm)	Absorption efficiency Q_{absPer}	Scattering efficiency Q_{scaPer}
20	Joint	499	1.346	0.3536	502	1.193	0.2835
80	Joint	484	1.451	1.485	489	1.432	1.486
140	Joint	459	1.288	1.399	470	1.290	1.397
200	Joint	444	1.189	1.365	454	1.195	1.355

Table S5a: Si nanowires - Absorption resonances for parallel polarization

Nanowire radius r (nm)	Order of resonance n	Temperature, $T = 200$ K			Temperature, $T = 650$ K		
		Resonance wavelength $\lambda_{\text{res}}^{\text{abs}}$ (nm)	Absorption efficiency Q_{absPar}	Scattering efficiency Q_{scaPar}	Resonance wavelength $\lambda_{\text{res}}^{\text{abs}}$ (nm)	Absorption efficiency Q_{absPar}	Scattering efficiency Q_{scaPar}
20	zeroth	500	0.0944	4.142	500	0.2499	4.294
80	first	748	0.03445	5.859	778	0.1093	5.953
	second	554	0.2733	5.402	570	0.6513	4.609
140	Other	566	0.4744	2.364	582	0.5927	1.902
	Other	676	0.1828	3.316	694	0.4661	2.722
200	Other	648	0.3982	3.488	664	0.4603	3.060
	Other	754	0.1781	2.658	772	0.4221	2.239

Table S5b: Si nanowires - Absorption resonances for perpendicular polarization

Nanowire radius r (nm)	Order of resonance n	Temperature, $T = 200$ K			Temperature, $T = 650$ K		
		Resonance wavelength $\lambda_{\text{res}}^{\text{abs}}$ (nm)	Absorption efficiency Q_{absPer}	Scattering efficiency Q_{scaPer}	Resonance wavelength $\lambda_{\text{res}}^{\text{abs}}$ (nm)	Absorption efficiency Q_{absPer}	Scattering efficiency Q_{scaPer}
20	zeroth	500	5.583e-3	0.0873	500	1.604e-2	0.0925
80	first	543	0.1434	3.1105	560	0.3674	2.774
140	Other	508	0.5508	2.737	522	0.6893	2.200
	Other	582	0.2178	1.913	598	0.5214	1.538
200	Other	586	0.4513	3.356	602	0.5933	2.688
	Other	666	0.2225	2.914	682	0.5252	2.489

Table S6a: GaAs nanowires - Absorption resonances for parallel polarization

Nanowire radius r (nm)	Order of resonance n	Temperature, $T = 200$ K			Temperature, $T = 650$ K		
		Resonance wavelength λ_{res}^{abs} (nm)	Absorption efficiency Q_{absPar}	Scattering efficiency Q_{scaPar}	Resonance wavelength λ_{res}^{abs} (nm)	Absorption efficiency Q_{absPar}	Scattering efficiency Q_{scaPar}
20	zeroth	500	0.7956	3.31	500	0.9693	3.275
80	first	756	0.5224	4.907	810	0.7592	4.745
	second	552	1.216	2.698	586	1.267	2.491
140	other	672	0.9414	1.559	700	0.9975	1.376
	other	844	0.6536	3.764	908	0.7805	4.141
200	other	754	0.7167	1.369	784	0.7823	1.355
	other	844	0.6165	0.9189	950	0.7195	2.078

Table S6b: GaAs nanowires - Absorption resonances for perpendicular polarization

Nanowire radius r (nm)	Order of resonance n	Temperature, $T = 200$ K			Temperature, $T = 650$ K		
		Resonance wavelength λ_{res}^{abs} (nm)	Absorption efficiency Q_{absPer}	Scattering efficiency Q_{scaPer}	Resonance wavelength λ_{res}^{abs} (nm)	Absorption efficiency Q_{absPer}	Scattering efficiency Q_{scaPer}
20	zeroth	500	6.537e-2	0.0925	500	9.950e-2	0.1022
80	first	540	0.9036	1.709	570	0.9713	1.614
140	other	584	1.028	0.7575	608	1.050	0.7554
200	other	784	0.6575	1.125	814	0.8743	0.904
	first	668	0.9741	1.249	696	0.9937	1.261
	second	584	0.9903	1.538	608	1.009	1.397
